# The Curious Incidence of Mucinous Adenocarcinoma Masquerading as Perianal Hidradenitis Suppurativa

**DOI:** 10.7759/cureus.57585

**Published:** 2024-04-04

**Authors:** Hafiz Nasir, Nabil Mohammad Azmi, Diana Melissa Dualim, Zairul Azwan Azman, Nur Afdzillah Abdul Rahman

**Affiliations:** 1 Department of Surgery, Faculty of Medicine, The National University of Malaysia, Kuala Lumpur, MYS

**Keywords:** pelvic radiotherapy, abdominoperineal resection, perianal mucinous adenocarcinoma, fistula in ano, hidradenitis suppurativa

## Abstract

Hidradenitis suppurativa (HS), also known as acne inversa, is a chronic inflammatory disorder affecting the terminal follicular epithelium within the apocrine skin glands. When these lesions develop in the genital and perianal regions, there is a potential risk of progression to squamous cell carcinoma or mucinous adenocarcinoma. The tumor may appear in the perianal area, perineum, or buttocks. Here, we present a rare case of long-standing perianal HS with associated fistula-related mucinous adenocarcinoma and the challenges we faced in managing this condition.

## Introduction

Perianal mucinous adenocarcinoma (PMA) can be a great mimic and pose a challenge in making a diagnosis. They may present as Hidradenitis suppurativa (HS), which is a benign, chronic inflammatory disorder affecting the terminal follicular epithelium within the apocrine skin glands. When these lesions develop in the genital and perianal regions, there is a potential risk of progression to squamous cell carcinoma or mucinous adenocarcinoma [1]. The tumor may appear in the perianal area, perineum, or buttocks [2]. Here, we present a rare case of long-standing perianal HS with associated fistula-related mucinous adenocarcinoma.

## Case presentation

**Figure 1:**
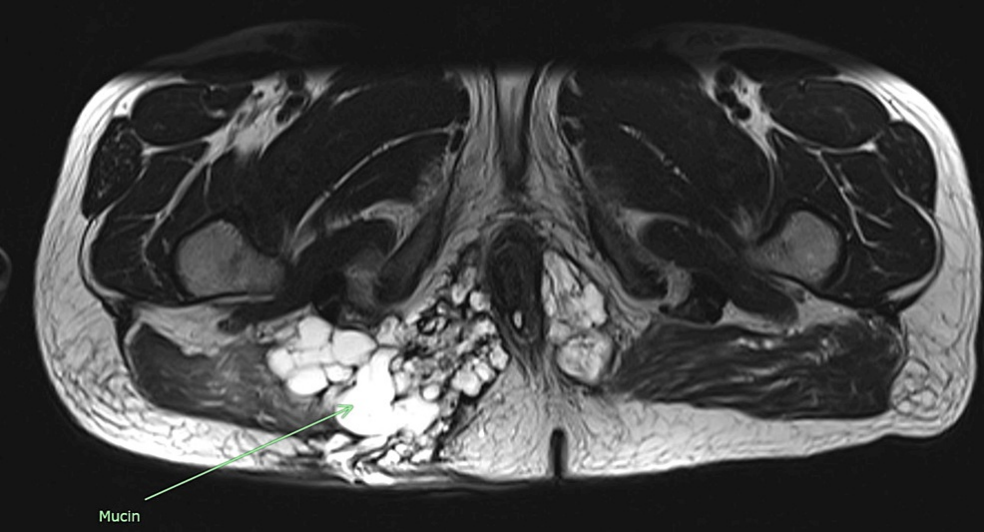
The MRI (coronal section) showed a complex, multiloculated collection with a complex fistula seen communicating with the right buttock.

**Figure 2:**
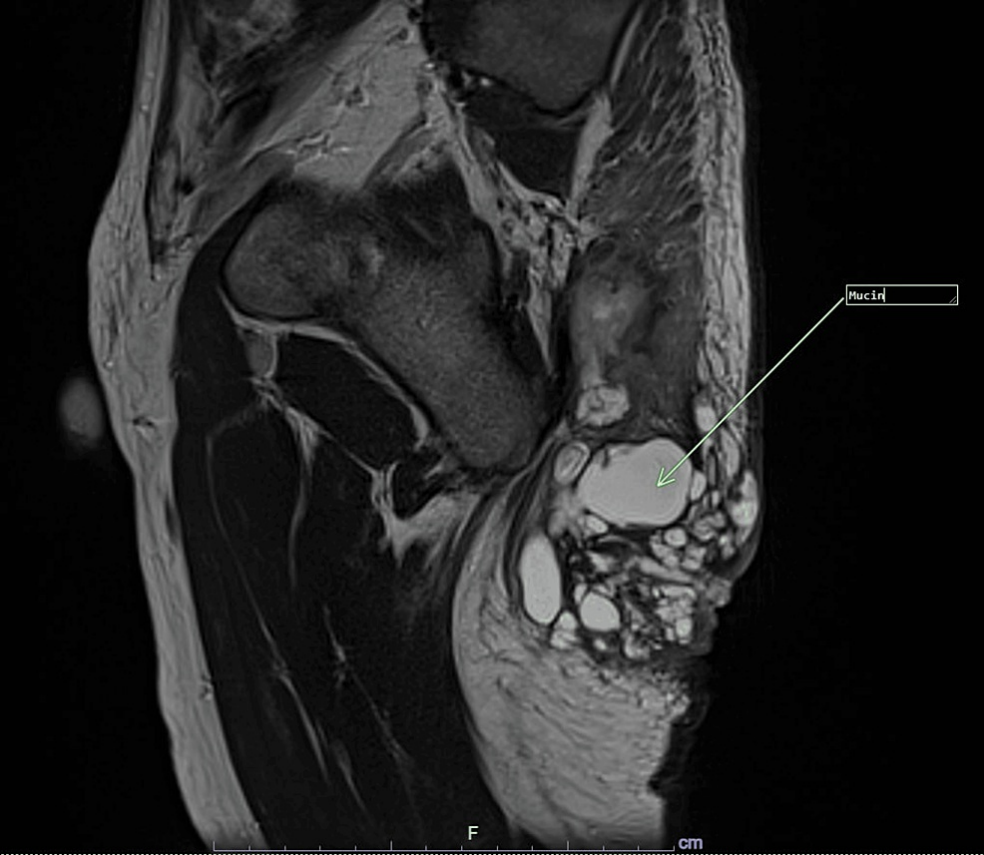
MRI (sagittal section): A complex, multiloculated collection containing mucin communicating with the right buttock was demonstrated.

A 53-year-old man presented with a prolonged history of multiple bilateral perianal wounds extending to the right gluteal region over the past decade. His treatment journey was challenging, marked by numerous episodes of surgical incision and drainage for perianal abscesses, together with Adalimumab biologics and antibiotics. Additionally, he underwent multiple insertions of draining setons for a fistula in ano. Due to persistent perianal disease, he was referred to our center for further management. At the initial presentation, there were no signs of sepsis, as he was afebrile with normal inflammatory markers. The white blood cell (WBC) was 10.5 x 109/L and the c-reactive protein (CRP) was 1.0 x mg/L. Tumor marker carcinoma embryonic antigen (CEA) was within the normal range of 2.5 ng/mL. Examination revealed no purulent discharge from the perianal wound but a complicated fistula in the ano accompanied by perianal (HS) extending to the right gluteal region. Multiple external openings were noted, along with an internal opening at the 7 o'clock position. Magnetic resonance imaging (MRI) of the pelvis depicts HS complicated by a perianal abscess and a complex fistula in ano, as illustrated in Figures 1, 2. 

**Figure 3:**
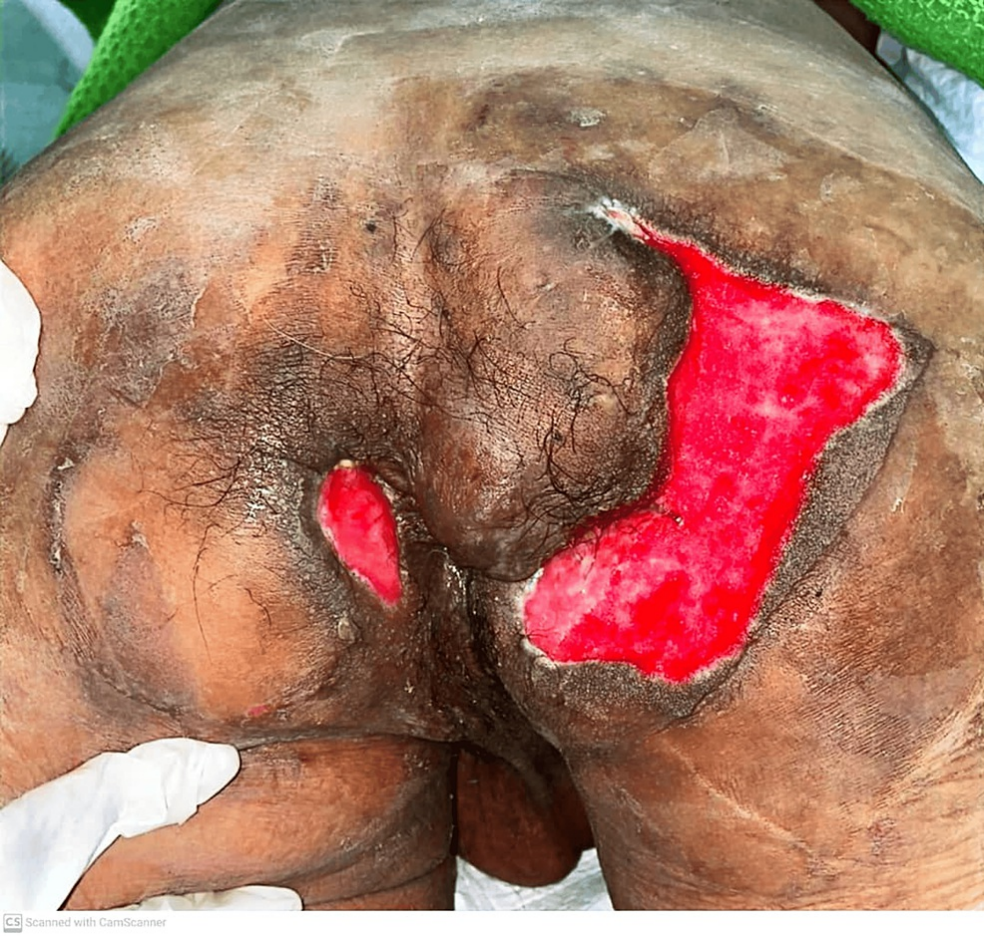
Evidence of granulating wounds after excision and regular wound dressing.

**Figure 4:**
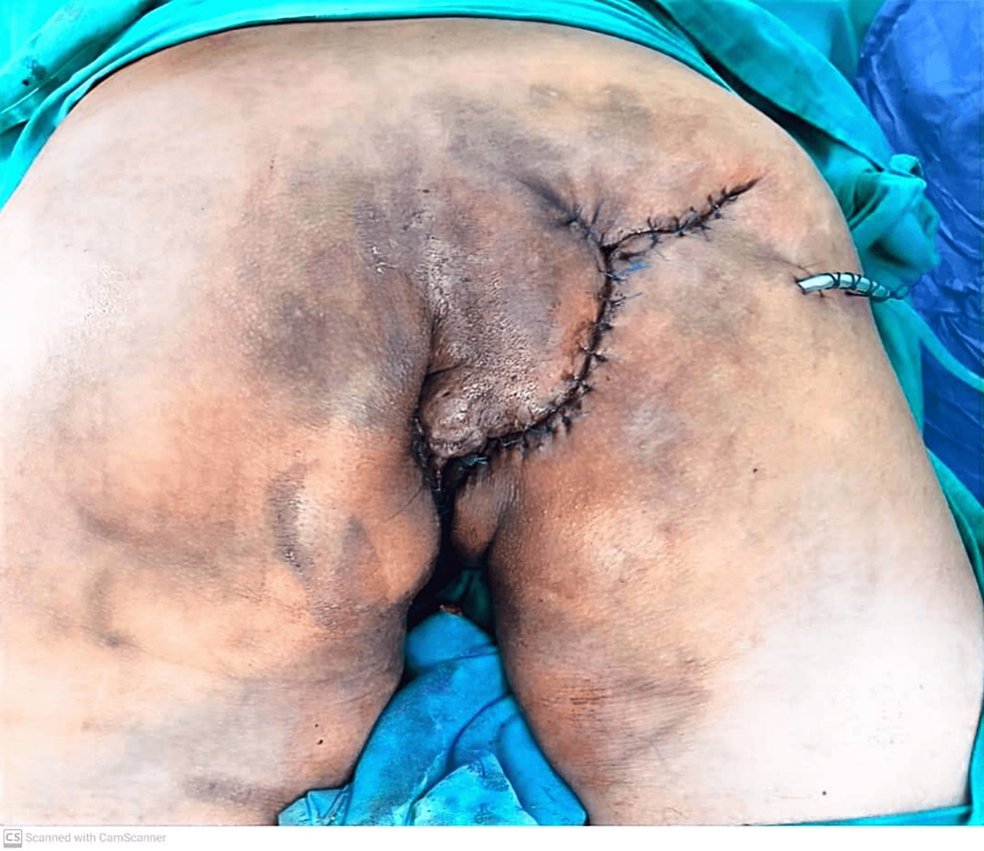
Wound closure by local flap.

The patient underwent examination under anesthesia and an incisional biopsy, revealing low-grade dysplasia with no signs of malignant transformation on histopathological examination. A surgical intervention was planned and executed, involving wide local excision of tissue from the right gluteal area and bilateral perianal regions, along with wound debridement, fistulectomy, and insertion of a seton. Additionally, a laparoscopic-assisted diversion loop sigmoid colostomy was performed to aid in wound management. Subsequently, the patient required multiple wound dressings and transitioned to a vacuum-assisted dressing once the wound bed had granulated. Closure of the wound was achieved through the utilization of a local flap one month post-operation, as depicted in Figures 3, 4.

**Figure 5:**
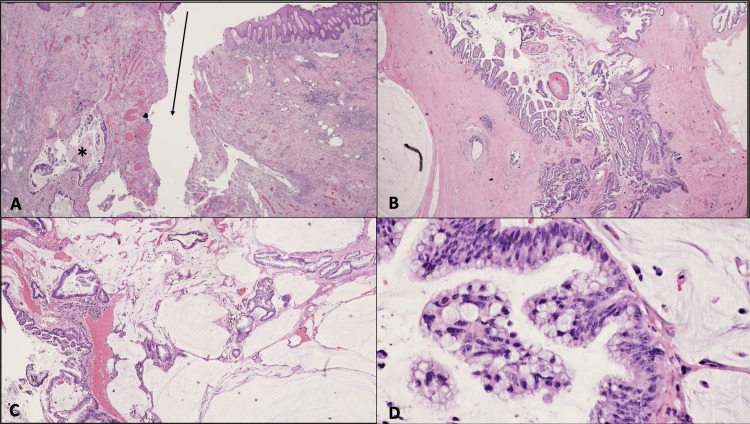
(A) The anal fistula (arrow) is lined by granulation tissue with an adjacent focus of mucinous carcinoma (asterisk) (H&E, 1x). (B) The tumor forms glandular and villous-like structures (H&E, 4x). (C) Malignant glands are also observed within extracellular pools of mucin (H&E, 1x). (D) The malignant cells show hyperchromatic nuclei, small nucleoli, and apical mucin (H&E, 40x).

The histopathological examination yielded unexpected findings, revealing a fistula-associated mucinous adenocarcinoma categorized as pT3, with involvement of both peripheral and deep margins. No indications of lymphovascular or perineural invasion were observed. Further details regarding this result are provided in Figure 5. 

The unexpected result prompted a multidisciplinary team discussion (MDT) involving oncologists and radiologists to reach a consensus on further treatment. The outcome of the MDT consensus was to commence adjuvant radiotherapy for local disease control. Additionally, the patient will undergo surveillance follow-up, including a colonoscopy and pelvic MRI. The patient was counseled regarding these measures and agreed to undergo a salvage abdominoperineal resection (APR) with flap closure if the tumor recurs.

## Discussion

Perianal mucinous adenocarcinomas (PMAs) are uncommon, accounting for only 2-3% of gastrointestinal malignancies [3,4]. PMA can mimic benign inflammatory perianal conditions like long-standing hidradenitis suppurativa and perianal Crohn’s disease, posing challenges in diagnosis [5]. They tend to be locally invasive, frequently leading to complications such as fistulas in ano, as observed in this particular case. Both perianal Crohn's and colorectal adenocarcinoma are important diagnoses to consider and have been ruled out through blood investigations and by colonoscopy and biopsy.

The diagnostic criteria for perianal mucinous adenocarcinoma require the fistula to precede the carcinoma, the exclusion of synchronous colorectal carcinoma, and confirmation that the internal opening of the fistula connects to the anal canal rather than the malignancy [6]. In this case, the patient fulfills all three criteria. Diagnosis often experiences delays because these lesions mimic symptoms of benign anorectal inflammatory conditions, and accurately identifying the tumor through biopsies can be challenging and prone to inaccuracy [7]. The pathogenesis remains uncertain, with some literature suggesting that carcinomatous cells originate from anal ducts and glands. Long-standing inflammation, friction, and scarring of the perianal area have been proposed as potential causes of malignant transformation in these cells [8]. In this case, CEA was within normal limits, and this may occur in colorectal adenocarcinomas.

Diagnosis demands a high level of suspicion, often leading clinicians to utilize various imaging techniques. These may include endoanal ultrasound, computed tomography scans (CT scans), and magnetic resonance imaging (MRI) of the pelvis. A contrasted CT scan of the thorax, abdomen, and pelvis showed no distant metastasis. In our practice, MRI has proven to be the most effective method for diagnosing and detailing the anatomy of the lesion. It can precisely depict the perianal region's anatomy, including the presence of fistulas, abscesses, mucin content, and involvement of perianal structures such as muscles and the anal canal. In this specific case, the immunohistochemistry technique was utilized to ascertain the origin of the carcinoma, commonly employing cytokeratins (CKs), which are intermediate-sized filament proteins in the cytoskeleton. In a case series by Azeddine Diffaa et al., the three primary anal mucinous adenocarcinomas showed positivity for CK7 and negativity for CK20. In our case, both CK7 and CK20 were found to be positive.

The rarity of these tumors and the absence of controlled clinical trials contribute to the lack of consensus regarding their treatment. Presently, the most recommended approach involves wide local excision for locoregional control. Following surgery, radiotherapy with or without chemotherapy is considered an option, although the outcomes remain subject to debate [9]. According to Natalia et al.'s literature review, a total of five cases of mucinous adenocarcinoma in individuals with chronic suppurative hidradenitis were identified [10]. All patients underwent extended abdominoperineal resection (APR). Among these cases, two received adjuvant radiotherapy, while the remaining three received adjuvant chemotherapy and radiotherapy. However, in this specific case, we chose to perform a wide local excision of the hidradenitis suppurativa (HS) after the initial incision biopsy showed low-grade dysplasia only. 

Managing this case post-surgery presents a significant challenge due to the potential high rate of recurrence. A multidisciplinary team meeting involving radiologists and oncologists was convened to determine the best course of action. Following consensus, the patient was offered adjuvant radiotherapy to decrease the likelihood of locoregional recurrence. A total dose of 40 Gy of five fractions per week of pelvic irradiation was given. Surveillance will involve monitoring carcinoembryonic antigen (CEA) levels, conducting endoanal ultrasound, and utilizing MRI scans.

The management of this case underscores the importance of a multidisciplinary team (MDT) to achieve optimal outcomes in handling complex cases. The patient was subsequently counseled regarding the potential need for salvage APR if the disease recurs. This surgical approach is advocated in various literature. However, it is extensive and results in a large wound defect necessitating flap coverage. The Vertical Rectus Abdominis Myocutaneous (VRAM) flap appears to be the preferred choice of flap, according to Ilan Kent et al. [9]. This retrospective observational study demonstrated that the VRAM flap has minimal perineal wound complications and a low incidence of perineal wound hernias. Perineal wound complications range from 0-30%, particularly higher in salvage APR cases following radiotherapy. The VRAM flap offers several advantages, including providing large, voluminous, well-vascularized tissue capable of covering extensive perineal defects. This flap is based on the epigastric artery and vein and can reach defects up to 25cm from the groin [11].

## Conclusions

In conclusion, PMA gives rise to a significant challenge in making a diagnosis and necessitates a high level of suspicion. Initial biopsy results can be misleading, and subsequent treatment often involves a multimodal approach, including radiotherapy and salvaging APR in cases of disease recurrence. This underscores the complexity of managing PMA and the importance of a comprehensive and multidisciplinary approach to achieve optimal patient outcomes. This case elucidates the reality that surgeons and physicians face when managing complex cases.
